# Supplementary material for: Macrophage Sult2b1 promotes pathological neovascularization in age-related macular degeneration
Source: Life Sci Alliance. 2023 Aug 7;6(11):e202302020. doi: 10.26508/lsa.202302020 (PMC10427760; doi:10.26508/lsa.202302020)

Fig.3E

anti-SULT2B1

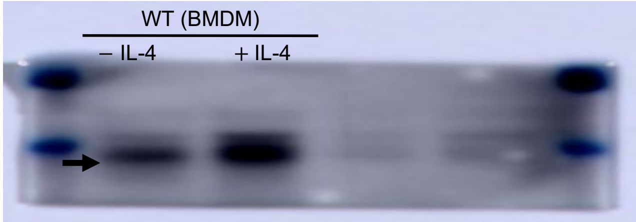

anti-GAPDH

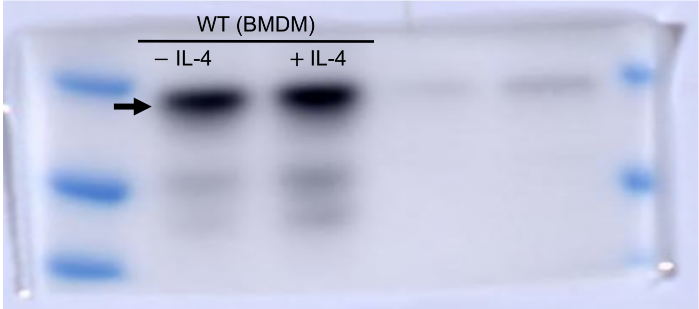

Fig.3F

anti-YM1

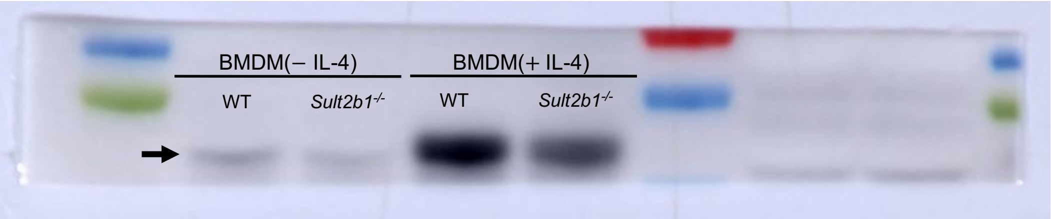

anti-ARG1

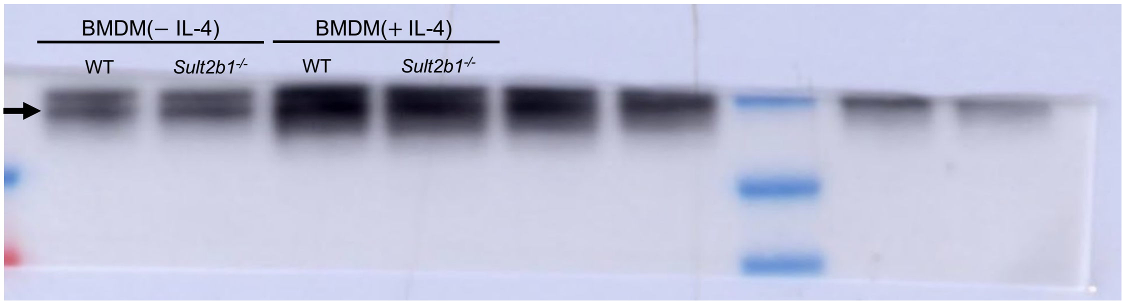

anti-ACTIN

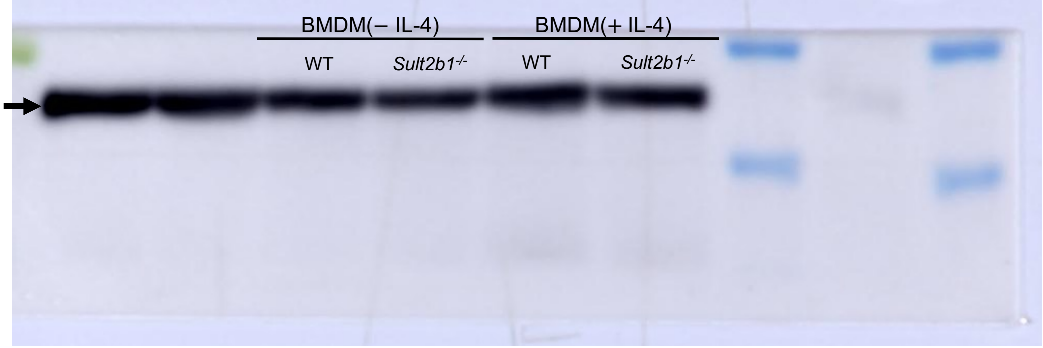

Fig.4E

anti-LXRα

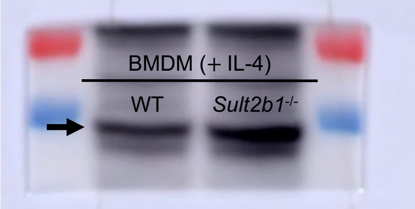

anti-LXRβ

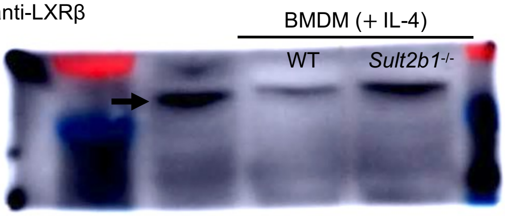

anti-ABCA1

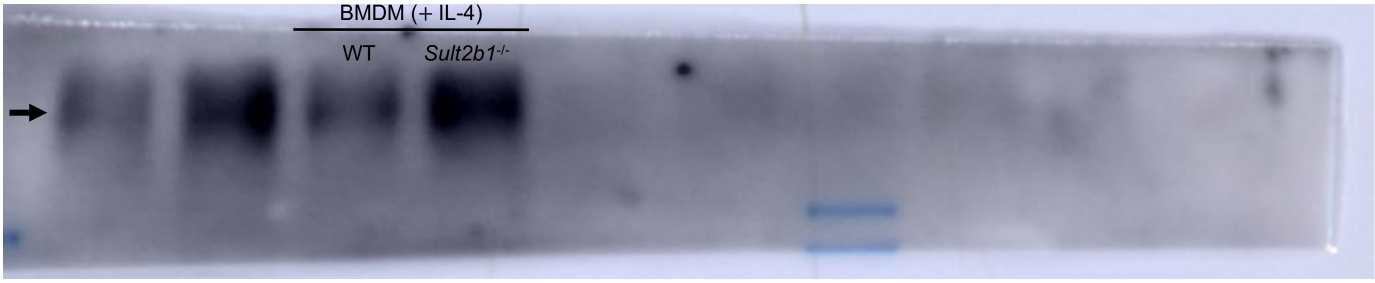

anti-ABCG1

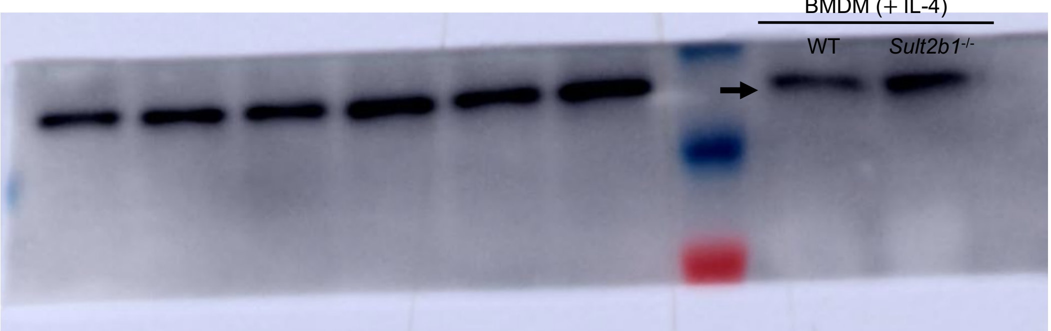

anti-GAPDH

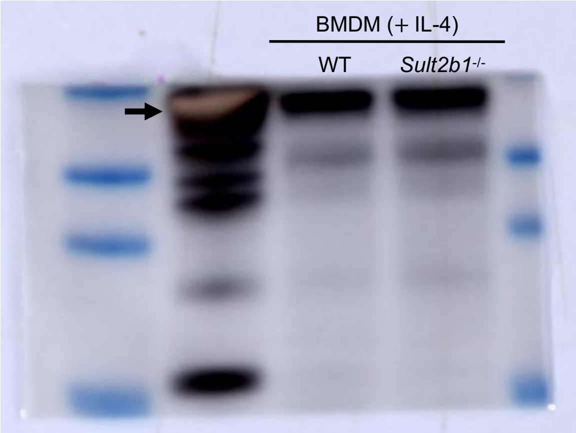

Fig.6A

anti-LXRα

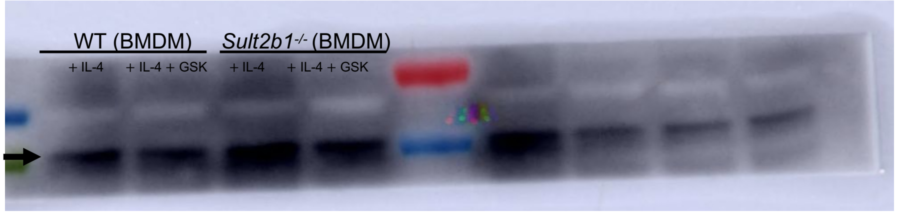

anti-LXRβ

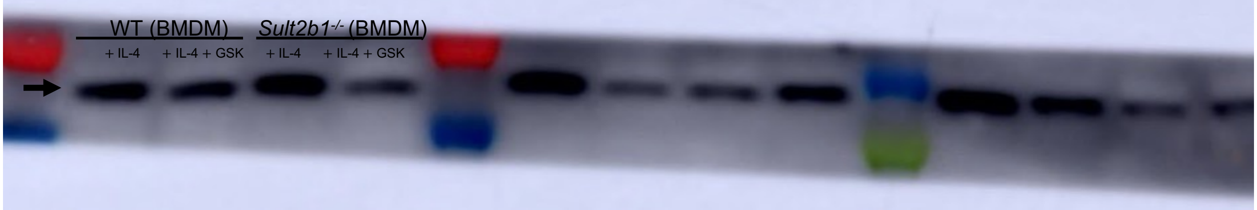

anti-ABCA1

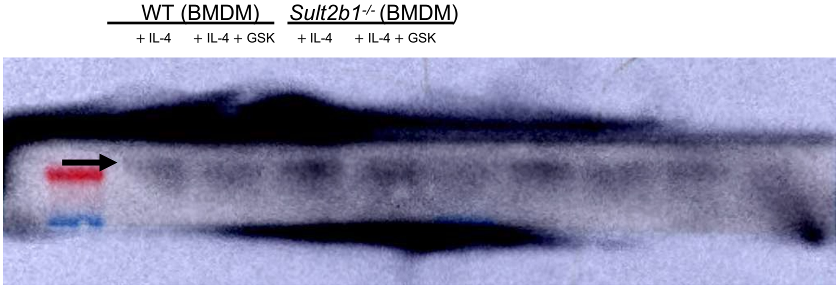

anti-ABCG1

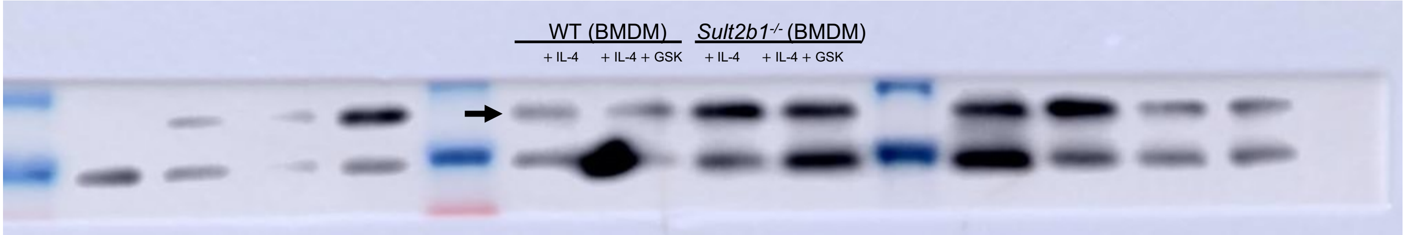

anti-GAPDH

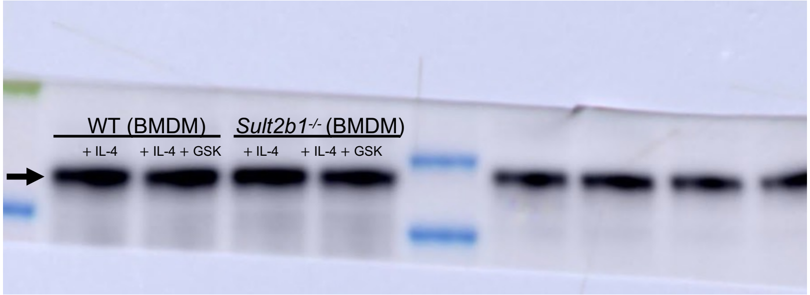

Fig.6C

anti-YM1

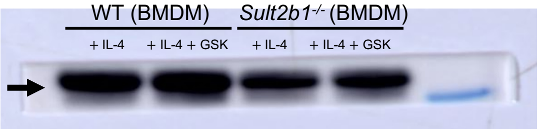

anti-ARG1

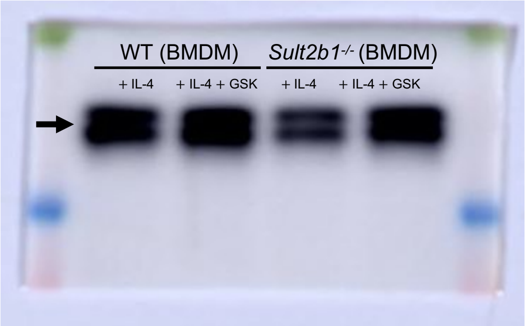

anti-GAPDH

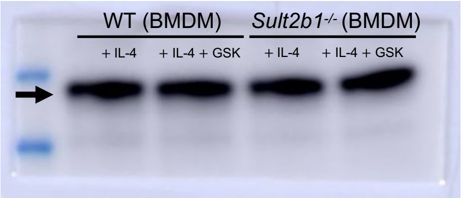

Supplement: Supplementary file 1 [file LSA-2023-02020_SdataF3_F4_F6.pdf]
