# Supplementary material for: Macrophage Sult2b1 promotes pathological neovascularization in age-related macular degeneration
Source: Life Sci Alliance. 2023 Aug 7;6(11):e202302020. doi: 10.26508/lsa.202302020 (PMC10427760; doi:10.26508/lsa.202302020)
Supplement: Supplementary file 2 [file LSA-2023-02020_TableS1.docx]

**Table S1** Genotyping primers.

| **Primer** | **Sequence (5’-3’)** |
| --- | --- |
| ***Sult2b1***-Forward primer 1 | CTTATTCAACCACCACACCCAT |
| ***Sult2b1***-Reverse primer 2 | TCCATCCCTAGCTTCACATGG |
| ***Sult2b1***-Reverse primer 3 | GACAGCGCAGGGCCACAC |
| ***Sts***-Forward primer | AGGGGGAAGGCCATGAGTACA |
| ***Sts***-Reverse primer | ACTTCCTGTCCGTCTGACCTCAGT |
