# Supplementary material for: Macrophage Sult2b1 promotes pathological neovascularization in age-related macular degeneration
Source: Life Sci Alliance. 2023 Aug 7;6(11):e202302020. doi: 10.26508/lsa.202302020 (PMC10427760; doi:10.26508/lsa.202302020)
Supplement: Supplementary file 3 [file LSA-2023-02020_TableS2.docx]

**Table S2** The primers used for the polymerase chain reaction (PCR) reaction.

| **Primer** | **Sequence (5’-3’)** |
| --- | --- |
| ***Gapdh***-Forward | ATCAAGAAGGTGGTGAAGCA |
| ***Gapdh***-Reverse | AGACAACCTGGTCCTCAGTGT |
| ***Sult2b1***-Forward | TCCTGTCGGCATGTACTCAC |
| ***Sult2b1***-Reverse | CGCACGTTGCTAGTGTTCTC |
| ***Vegfa***-Forward | ACTGGACCCTGGCTTTACTG |
| ***Vegfa***- Reverse | TCTGCTCTCCTTCTGTCGTG |
| ***Pdgfb***-Forward | CGGCCTGTGACTAGAAGTCC |
| ***Pdgfb***-Reverse | GAGCTTGAGGCGTCTTGG |
| ***Fgf2***-Forward | CGGCTCTACTGCAAGAACG |
| ***Fgf2***-Reverse | TGCTTGGAGTTGTAGTTTGACG |
| ***Tek***-Forward | CATAGGAGGAAACCTGTTCACC |
| ***Tek***-Reverse | GCCCCCACTTCTGAGCTT |
| ***Tgfb1***-Forward | TGGAGCAACATGTGGAACTC |
| ***Tgfb1***-Reverse | CAGCAGCCGGTTACCAAG |
